# Infrapatellar fat pad as a source of biomarkers and therapeutic target for knee osteoarthritis

**DOI:** 10.1186/s13075-025-03517-8

**Published:** 2025-04-05

**Authors:** Betzabeth Pereira Herrera, Kaj Emanuel, Pieter J. Emans, Martijn van Griensven, Berta Cillero-Pastor

**Affiliations:** 1 Department of Cell Biology-Inspired Tissue Engineering, MERLN Institute for Technology-Inspired Regenerative Medicine, University of Maastricht, Maastricht, The Netherlands; 2 Department of Orthopedic Surgery and Sports Medicine, Amsterdam UMC, Location AMC, University of Amsterdam, Amsterdam Movement Sciences, Amsterdam, The Netherlands; 3 Department of Orthopedic Surgery, Joint-Preserving Clinic, Maastricht University Medical Center+, Maastricht, The Netherlands; 4 Maastricht MultiModal Molecular Imaging Institute (M4I), Maastricht University, Maastricht, The Netherlands

**Keywords:** Biomarkers, Diagnostic, Infrapatellar fat pad, MRI, Osteoarthritis, Prognostic, Proteomics, Transcriptomics

## Abstract

**Background and objective:**

Osteoarthritis (OA) is a multifactorial and highly prevalent disease in elderly adults; however, its pathogenesis, diagnosis, and treatment are unmet needs nowadays. Research efforts have focused on elucidating the molecular mechanisms involved in the pathogenesis, onset, and progression of OA to facilitate early detection and effective therapeutic approaches. Infrapatellar fat pad (IPFP) represents a promising novel source of OA biomarkers given that it is an active player in OA. This review aims to investigate the current literature regarding the potential of the IPFP as a source of diagnostic and prognostic biomarkers for OA as well as potential target for novel therapies.

**Methods:**

A literature search was conducted in the PubMed database in June 2024. We included cross-sectional and longitudinal studies based on IPFP from human OA patients, oriented in the identification of imaging, biochemical, and molecular biomarkers in the IPFP.

**Results:**

After screening and evaluation, we included a total of 61 studies. Most of the imaging publications (*n* = 47) on IPFP are based on magnetic resonance imaging (MRI) that revealed potential semiquantitative and quantitative imaging biomarkers linked to inflammation, fibrosis, pain, and joint degeneration imaging parameters. Biochemical and molecular studies (*n* = 14) pointed out an increase in interleukin-6 (IL-6), fatty acid-binding protein 4 (FABP4), adiponectin, and lysophosphatidylcholine (LysoPC) in the IPFP during OA progression.

**Conclusions:**

Imaging, biochemical, and molecular studies indicate OA potential biomarkers in the IPFP related to inflammation, lipid dysregulation, and fibrosis. The combination of imaging and biochemical biomarkers could provide a better prediction of OA onset and the identification of OA progressors at an early stage. The IPFP study could also reveal potential therapeutic targets with the vision of better precision medicine.

**Supplementary Information:**

The online version contains supplementary material available at 10.1186/s13075-025-03517-8.

## Introduction

Osteoarthritis (OA) is one of the most common musculoskeletal disorders, highly prevalent in adults over 55 years old, that leads to pain and disability [1–4]. OA is a whole-joint disease, characterized by articular cartilage degradation, chondrocyte hypertrophy, bone remodeling, osteophyte formation, and synovial inflammation [3]. Despite OA pathogenesis remains unknown, it is hypothesized that the onset of OA is linked to an imbalance in joint loading that affects the biology and structure of cartilage promoting its degradation and, in some patients, knee structure is rapidly degraded (OA progressor individuals) [4]. Factors such as obesity, traumatic knee injuries and reduced musculoskeletal fitness can also contribute to early OA [4–6].

Currently, OA is typically diagnosed by means of radiography along with patient symptoms in an advanced stage when the chances of slowing down or reverting its symptoms are reduced [7]. While total knee arthroplasty (TKA) is still the only available treatment for end-stage OA [8], there is an urgent need to improve clinical diagnosis by detecting OA in the early stage and predicting its progression. As such, research efforts have focused on the search for OA biomarkers. A biomarker can be considered as a defined characteristic, a biomolecule, or a molecular fragment that is released or expressed in response to pathological, or pharmacological processes [9]. Techniques such as magnetic resonance imaging (MRI) have improved the characterization of anatomical abnormalities within the joint, at earlier OA stages, providing potential imaging biomarkers [10]. Along with this, research carried out in synovial fluid, cartilage, and synovium has revealed a handful of potential biochemical biomarkers that have even been detected in biological fluids like serum and urine through non-targeted approaches (OMICS techniques) [11, 12]. Despite these advances, there is still no consensus on OA biomarkers [11–14].

**Fig. 1:**
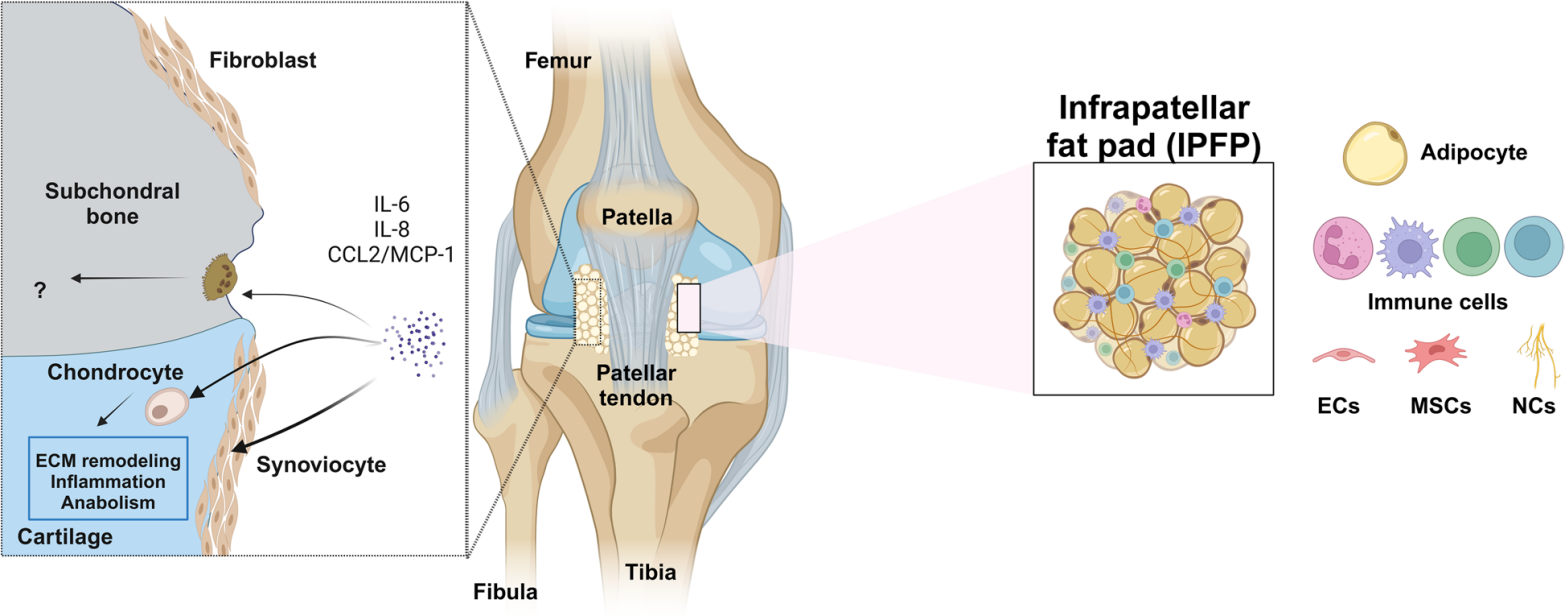
IPFP composition and proposed interaction between IPFP and other knee tissues. IPFP is mainly composed of adipocytes but can also hold immune cells, endothelial cells (ECs), mesenchymal stromal cells (MSCs), stem cells, and neuronal cells (NCs) (right). IPFP secretes pro-inflammatory mediators that induce extracellular matrix (ECM) remodeling and inflammation in chondrocytes [15], synoviocytes [16], and fibroblasts [25]. However, it has been reported that IPFP promotes cartilage anabolism [26] or protects from cartilage damage [20] (left). Created with Biorender

In this context, the infrapatellar fat pad (IPFP) has gained attention in recent years as a promising novel source of OA biomarkers given its concomitant inflammation may aggravate joint damage [15, 16]. The IPFP, also known as Hoffa’s fat pad, is located between the capsular layer and the synovium, beneath the patella and above the tibia [17]. IPFP is mainly composed of adipocytes, immune cells, endothelial cells, neuronal cells, and stem cells. IPFP is involved in secretion of paracrine factors, vascularization, innervation, and immunological roles that could affect surrounding tissues [17, 18] (Fig. 1). Conversely, a protective role has been attributed to the IPFP due to the presence of mesenchymal stromal cells (MSCs) [19, 20]. Additionally, IPFP may contribute to the absorption of mechanical shocks and force distribution in the joint [21], function that is presumably impaired due to an altered connective tissue during OA [22, 23]. Notably, it is available in the clinic because it is partially or totally removed to improve visualization during knee surgery [24]. This makes the IPFP a promising tissue to screen patients at risk for early onset and those that could rapidly evolve to advanced stages. This review aims to investigate the potential of IPFP as a source of diagnostic and prognostic biomarkers for OA with special focus on imaging and omics techniques for biomolecular analysis.

## Methods

A literature search was conducted in the PubMed database on 4 June 2024 for studies that evaluated the IPFP through imaging, biochemical, and molecular techniques. The search included the following terms: ‘‘infrapatellar fat pad’’; ‘‘Hoffa’s fat pad’’; ‘‘osteoarthritis’’; ‘‘biomarker’’; ‘‘imaging biomarker’’; ‘‘imaging’’; ‘‘magnetic resonance imaging’’; ‘‘molecular biomarker’’; ‘‘mass spectrometry’’; ‘‘proteomics’’; ‘‘metabolomics’’; ‘‘lipidomics’’; ‘‘gene expression’’; and ‘‘RNA’’. Details of the complete search can be found in the Supplementary Table 1.

The inclusion criteria established were: 1) studies based on IPFP from human OA patients; 2) the study of IPFP by imaging, biochemical, and molecular techniques; 3) cross-sectional and longitudinal imaging studies; 4) differences between OA patients and control non-OA individuals including healthy, cadaveric donors or patients suffering knee injuries; 5) longitudinal studies; 6) full-text studies written in English. Exclusion criteria were: 1) case reports; animal-based studies, and reviews; 2) full-text not available or abstracts only; 3) no control groups in cross-sectional imaging-based studies and those based on biochemical and molecular techniques; 4) studies focused on other knee compartments; 5) studies based only on clinical evaluation without any imaging, biochemical, or molecular assessment; 6) studies focused on other pathologies; 7) studies not related with biomarker discovery (focused on OA treatment, MSCs for regenerative medicine, cell characterization).

## Results

**Fig. 2:**
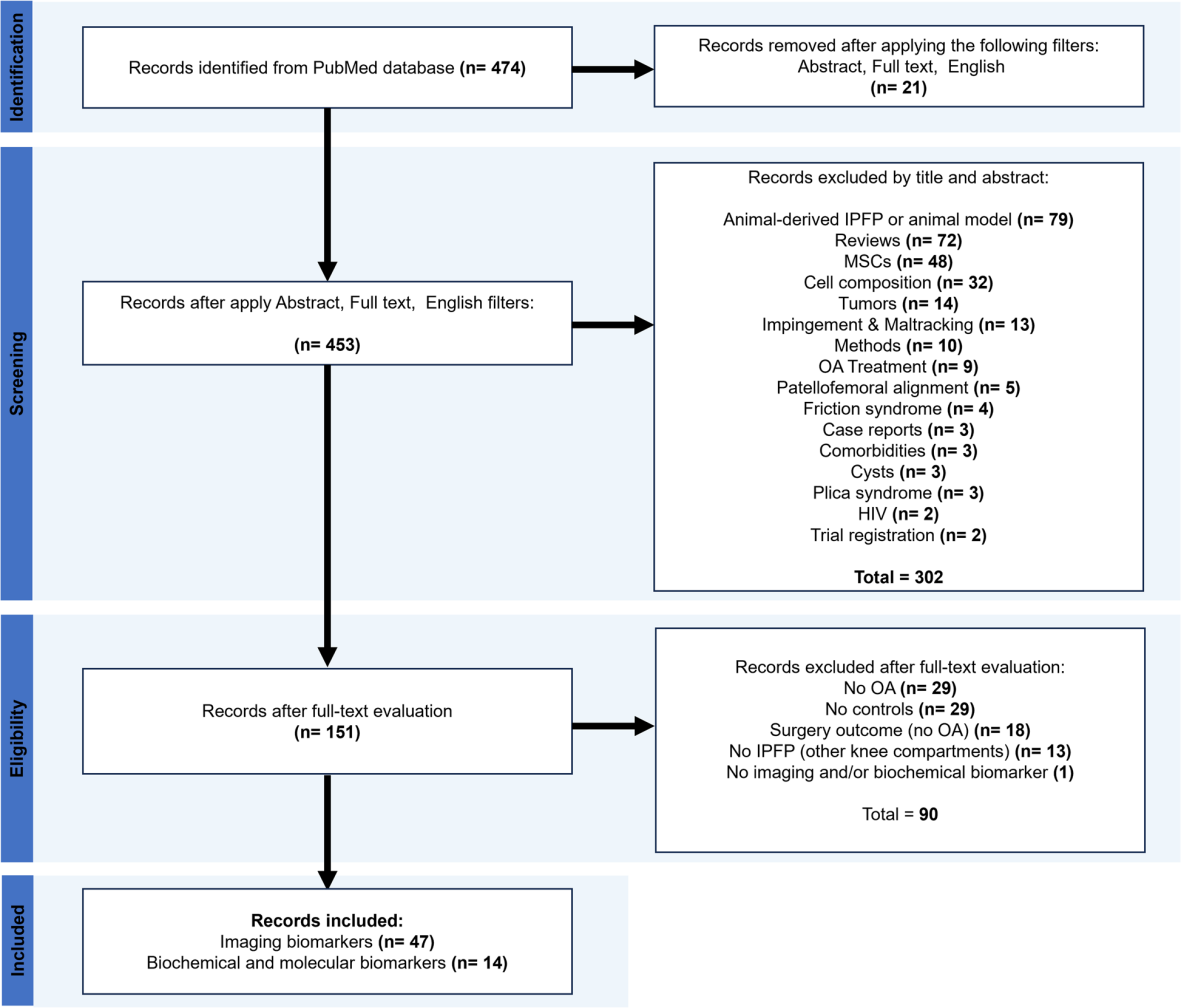
Flow diagram summarizing the literature search carried out in this work

The search in PubMed generated a collection of 474 records stored in NCBI. Then, we applied the following filters: ‘Abstract’, ‘Full text’, and ‘English’ as part of the inclusion criteria and 21 records were excluded. Subsequently, 453 records were loaded into EndNote 20.3 software and were screened by title and abstract, against the remaining inclusion and exclusion criteria, excluding 302 records. Finally, we evaluated the full-text of 151 records for eligibility and excluded 90 records. A total of 61 articles regarding imaging (*n* = 47) and biochemical/molecular (*n* = 14) IPFP biomarkers were included in this review (Fig. 2).

### Imaging markers

Forty-seven imaging studies included 11,142 individuals, from which 9714 were defined as OA, whereas 1428 were considered controls. Most OA patients were defined as radiographic (rOA) or detectable OA based on the Kellgren Lawrence (KL) grade system (KL ≥ 2). Other criteria to classify OA were, the American College of Rheumatology (ACR), joint space narrowing (JNS) score and/or the Osteoarthritis Research Society International (OARSI) atlas scale, and the Outerbridge score. The time frame for longitudinal studies varied from 0.5 to 5 years. On the other hand, control individuals were defined by those authors for having a KL < 2, or those with no progression when KL was < 2 over time. Other terms used comprised no rOA, or asymptomatic OA, healthy, patients with cruciate ligament, meniscal injuries, or patellofemoral pain. Demographic differences between control and OA patients were reported. Six studies found age-related differences, while four studies found those linked to body mass index (BMI). Most of the clinical research about the IPFP has arisen from MRI evaluations. The next section describes imaging parameters, and the findings reported for OA patients (Table 1).

**Table 1:** Potential imaging biomarkers in the IPFP from OA patients. (+) and (-) indicate positive or negative association, respectively. AUC: Area under curve. CSA: Cross-sectional area. FSE: Fast spin echo. IPFP [H]: High IPFP signal intensity alteration. IVIM-DWI: Intravoxel incoherent motion diffusion-weighted MR imaging. KOOS: Knee injury and osteoarthritis outcome score. MAVRIC: Multiacquisition variable-resonance image combination. MEDIC: Multi-echo data image combination. ML: Machine learning. OARSI: Osteoarthritis Research Society International. PD-w: Proton density-weighted. PFJ: Patellofemoral joint. rOA: Radiographic OA. SGE: Spoiled gradient echo. SPAIR: Spectral attenuated Inversion recovery. SPGR: Spoiled gradient recall. SWE: Ultrasound shear elastography. sOA: Symptomatic OA. TKA: Total knee arthroplasty. TSE: Turbo spin echo. T1-w: T1-weighted. T2FS: fat-suppressed T2 hyperintense regions T2-w: T2-weighted. WOMAC: Western Ontario and McMaster universities osteoarthritis Index. WORMS: Whole-organ MRI score. ^1^H-MRS: Hydrogen proton magnetic resonance spectroscopy

Reference	Population	Approach or MRI sequence	Findings
1) Cowan et al. [28]	Radiographic sOA, KL ≥ 2 (*n* = 35) Asymptomatic control (*n* = 11)	Fat-suppressed T2-w MEDIC	IPFP volume in symptomatic group
2) Cai et al. [29]	rOA, KL ≥ 2 (*n* = 174)	Fat-saturated T1-w 3-D SPGR Fat-saturated T2-w 2D FSE	(+) IPFP volume and cartilage volume (+) IPFP volume and BML and osteophytes
3) Fontanella et al. [30]	End-stage OA (*n* = 28) Patients meniscal tear (*n* = 32) ACLR (*n* = 29)	Fat-suppressed T1-w and T2-w	IPFP volume, surface, depth, and tibial arch length in end-stage OA IPFP volume in ACLR group IPFP hypointense signal in end-stage OA and ACLR
4) Ruan et al. [31]	sOA, KL ≥ 2 (*n* = 149) MMP-13 ≤ (*n* = 75) MMP-13 > (*n* = 74)	Fat-saturated T1-w 3-D SPGR Fat-saturated T2-w 2D FSE	(-) Serum MMP-13 with IPFP and cartilage volume (+) Serum MMP-13 with KL grading, IPFP [H], cartilage defect, serum IL-8, IL-18, TNFα
5) Chuckpaiwong et al. [32]	OA, KL = 2–3 (*n* = 15) Control Healthy (*n* = 15)	Fat-suppressed T1-w 3D No-fat-suppressed T2-w 3D	No differences in IPFP volume OA IPFP volume increased with age
6) He et al. [33]	Clinical OA (*n* = 53) Control Healthy (*n* = 54) 21 vs 21 matched by age, BMI, gender	3D T1-w FSE 3D PD-w fat-suppressed FSE	No correlation between knee pain and IPFP volume or area (+) IPFP signal and cartilage loss (-) IPFP signal and total pain
7) Steidle-Kloc et al. [34]	rOA kl 2–3 (*n* = 46)	Fat-suppressed	No association between IPFP volume and knee pain
8) Tan et al. [36]	sOA, KL = 2–3, WOMAC ≥ 4 (*n* = 84) Asymptomatic OA, KL = 2–3 (*n* = 43) Control Healthy, KL = 0–1 (*n* = 30)	IVIM-DWI	IPFP depth in OA groups IPFP [H] in the sOA compared to the asymptomatic OA
9) Fontanella et al. [37]	Late OA, undergoing TKA (*n* = 12) Moderate OA, outerbridge score 3–4 undergoing meniscectomy (*n* = 15) Control meniscal tears outerbridge score 0 (*n* = 17)	Fat-suppressed T2-w	IPFP depth, femoral, and tibial length Hypointense signal in moderate and late OA
10) Liu et al. [38]	OA KL ≥ 2 (*n* = 68) Control KL = 0–1 (*n* = 41)	PD-w-SPAIR: T2-w TSE T1-w TSE	IPFP maximum CSA and IPFP depth in OA group ( +) IPFP [H] with age, meniscal injury, cartilage injury, and bone marrow edema
11) Han et al. [39]	OA (*n* = 977)	Fat-saturated T1-w 3D	(+) IPFP maximum area and cartilage volume (-) IPFP maximun area and rOA
12) Satake et al. [40]	OA KL ≥ 2 (*n* = 97) Patients with PFJ OA Presence anterior knee pain (*n* = 41) Absence anterior knee pain (*n* = 56)	SWE and MRI: Fat-saturated T1-w 3D Fat-suppressed T2-w 2D	(+) IPFP stiffness with anterior knee pain and femorotibial osteoarthritis (-) -IPFP size and femorotibial osteoarthritis
13) Wang et al. [41]	sOA KL ≥ 2 (*n* = 45) Control (*n* = 45) KL = 0–1	Fat-suppressed T2-w	(+) IPFP [H] and sOA
14) Carotti et al., [42]	Symptomatic OA (*n* = 149)	Fat-suppressed T1-w and T2-w	(+) WOMAC knee pain and IPFP synovitis
15) Ruan et al. [43]	rOA KL ≥ 2 (*n* = 160) IL-8 ≤ median (*n* = 81) IL-8 > median (*n* = 79)	Fat saturated T1-w 3D Fat saturated T2-w 2D	+) Serum IL-8, IPFP [H], and serum bone and/or cartilage biomarkers
16) Wang et al. [44]	rOA KL ≥ 2 (*n* = 170) IL-17 ≤ median (*n* = 85) IL-17 > median (*n* = 79)	Fat saturated T1-w SPGR Fat saturated T2-w FSE	(+) (IPFP [H] with serum resistin and IL-17
17) Han et al. [45]	sOA (*n* = 200)	Fat saturated T1-w SGE Fat-suppressed T2-w FSE	(+) Serum resistin with IPFP IPFP [H] and knee synovitis
18) Wu et al. [46]	sOA KL ≥ 2 (*n* = 146) Ghrelin ≤ median (*n* = 74) Ghrelin > median (*n* = 72)	Fat saturated T1-w 3D Fat saturated T2-w 2D	(+) Ghrelin quartiles with IPFP IPFP [H], MMP3 and MMP13
19) Bian et al. [47]	sOA KL ≥ 2 (*n* = 137) Citrate < median (*n* = 68) Citrate ≥ median (*n* = 69)	Fat saturated T2-w	(-) Serum citrate with IPFP [H]
20) de Vries et al. [51]	OA undergoing TKA KL ≥ 2 (*n* = 22) PFP (*n* = 35) Healthy (*n* = 43)	T2 and DCE-MRI	73% OA patients showed T2FS-hyperintense IPFP regions (+) IPFP T2FS-hyperintense regions with perfusion in OA patients
21) Han et al. [52]	OA (*n* = 874) OARSI atlas	Fat-suppressed T1- or T2-w	(+) IPFP hypointense signals and rOA (+) IPFP hypointense signals with cartilage defects and BMLs (longitudinal, 2.7 years)
22) Okita et al. [53]	OA KL = 1–4 (*n* = 15) Healthy (*n* = 8)	T1- 3D MRI	IPFP contracture in OA
23) Chen et al. [54]	Advanced OA KL = 3–4 (*n* = 20) Mild OA KL = 2 (*n* = 20) No OA KL = 0–1 (*n* = 20)	T1-w PD SPAIR 3D six echo GRE	FF and T2* in end-stage OA (-) FF and T2* and the BML, Hoffa-effusion synovitis, cartilage defect, total knee pain
24) Zhong et al. [55]	Advanced OA KL = 3–4 (*n* = 16) Mild OA KL = 2 (*n* = 25) Healthy KL < 1 (*n* = 23)	^1^H-MRS	(-) FF and OA severity and Hoffa-synovitis A weak inverse correlation with knee pain
			**Prognostic**
25) Ruhdorfer et al. [56] 2 years	OA KL = 1–3 (*n* = 110) Control no progression knees (*n* = 118) Healthy (*n* = 88)	Intermediate-w fat-suppressed FSE	IPFP [H] in progressor OA knees
26) Harkey et al. [57] 2 years	Accelerated OA KL from 0–1 to 3–4 (*n* = 113) No accelerated OA KL from 0–1 to 1–2 (*n* = 241)	Intermediate-w fat-suppressed TSE Intermediate-w TSE, 3D dual-echo steady-state	Patients with increased IPFP [H] had a higher probability of developing end-stage OA
27) Davis et al. [58] 2 years	Accelerated OA (*n* = 125) KL from 0–1 to 3–4 Common OA (*n* = 125) Control KL = 0–1 no changes in 4 years (*n* = 125)	Intermediate-w TSE fat-suppressed	IPFP [H] in end-stage OA compared to moderate OA, at 1 year before OA onset
28) Hill et al. [59] 0.5 years	rOA (*n* = 270)	Fat-suppressed T2-w SE, PD	(+) Pain and IPFP synovitis
29) Roemer et al. [60] 5 years	Severe OA KL = 3–4 (*n* = 125) No/mild OA KL ≤ 2 (*n* = 46)	Intermediate-w TSE 3D dual-echo the 3D dual-echo at steady-state Intermediate-w fat-saturated TSE	Hoffa synovitis was less frequent in No/mild rOA at baseline Hoffa synovitis was similar between severe and No/mild rOA before TKA
30) Lu et al. [61]	sOA KL ≤ 3 (*n* = 100)	Fat-saturated T2-w 3D SE	(+) IPFP sDev [H] and clustering factor [H], cartilage defect, bone marrow lesions and rOA
31) Wang et al. [62] 4 years	rOA KL ≥ 2 (*n* = 322) Control No rOA in 4 years (*n* = 355)	Intermediate-w T2-w TSE	(+) IPFP Median [H], UQ [H], and the clustering factor [H] with incident rOA (+) All measures with incident rOA 1 year prior OA detection
32) Cen et al. [63]	OA KL = 1–3 (*n* = 600)	Fat-saturated T2-w	(+) IPFP Mean [H] and Clustering factor [H] with radiographic and pain group (+) IPFP [H] and radiographic group compared with pain group
33) Wang et al. [64] 5 years	OA underwent TKA after 5 years (*n* = 127) Control no TKA after 5 years (*n* = 127)	Fat-saturated T2-w TSE	Association with TKA Baseline: (+) Percentage (H) 1 year before TKA: (+) sDev [H], Percentage [H], and Clustering factor [H] Before TKA: (+) all measurements
34) Han et al. [65] - 2 years	sOA (*n* = 261)	Fat-suppressed T2-w FSE	Baseline: (+) sDev [H], UQ [H], and clustering factor [H] with tibiofemoral cartilage defects, and loss of tibial cartilage volume
35) Ruan et al. [66]	OA KL ≤ 3 (*n* = 255)	T2-w	(+) sDev [H], UQ [H], percentage [H], and clustering factor [H] with effusion-synovitis
36) Cen et al. [67]	OA KL = 1–3 (*n* = 600)	Fat-saturated T2-w	(+) Mean [H], sDev [H], Median [H], UQ [H], Percentage [H] and cartilage degradation (uC2C, uCTX-II) bone turnover (uCTX-Iα and uNTX-I) (+) Mean [H], Median [H] and UQ [H] with bone turnover (sCTX-I and uCTX-Iβ) (+) Mean [H], Median [H]. and Percentage [H] with cartilage degradation (Coll2-1 NO2) (+) sDev [H], Percentage [H] and inflammation (sHA) No associations were found with Clustering factor [H]
37) Li et al. [69]	OA KL ≥ 2 4 years (*n* = 345) Control no OA after 4 years (*n* = 345)	Voxel-based texture MRI	Diagnostic performance (AUC, 0.75)
38) Ye et al. [71]	Detectable OA KL ≥ 2 (*n* = 130) No detectable OA KL < 2 (*n* = 34)	Radiomics	AUC of 0.78 in test datasets (+) rad-scores and WORMS of cartilage, bone, meniscus, ligament, and synovium
39) Yu et al. [72]	OA KL ≥ 2 (*n* = 302) Control KL = 0–1 (*n* = 302)	Radiomics	Diagnostic performance (AUCs, above 0.70)
40) Bonakdari et al. [73] -	OA patients (*n* = 678) High-BMI (*n* = 341) Low-BMI (*n* = 337)	ML	Best models to predict IPFP volume: gender, age, and BMI, combined with a) Total-cohort: adipsin/chemerin b) High-BMI: chemerin/adiponectin HMW c) Low-BMI: IL-8
			**Surgery outcome**
41) Sacher et al. [74]	TKA (*n* = 28)	MAVRIC	T2 values in subjects with severe IPFP scarring
42) Cankaya et al. [76]	TKA Total (*n* = 36) Partial (*n* = 36)	Clinical and Isokinetic	Worse isokinetic performance
43) Gwyn et al. [77]	TKA Total (*n* = 72) Partial (*n* = 39)	Radiography	Patellar tendon lenght
44) Pinsornsak et al. [78]	TKA (*n* = 90) Total (*n* = 45) Partial (*n* = 45)	Clinical and sonographic (radiology)	No differences in patellar tendon shortening, and knee functionality Anterior knee pain in resected group
45) İmren et al. [79] 5 years	TKA (*n* = 224)	Radiography	No differences in patellar tendon length
46) Michalak et al. [80] 0.5 years	TKA (*n* = 65)	Clinical and isokinetic	No differences in KOOS, functional outcomes, anterior knee pain, or patellar tendon length
47) Sellars et al. [81]	TKA (*n* = 111)	Radiography	No changes in patella tendon lenght

#### Morphological appearance

The IPFP morphology (volume, area, and depth) can be evaluated by MRI through manual, semi-automated or automated assessments [27]. Seven studies reported inconclusive results regarding IPFP volume, a parameter that is measured by tracing the fat boundary [27]. Increased IPFP volume was found in OA patients [28] linking positively with osteophytes, pain, and cartilage lesions [28, 29]. In contrast, other studies showed a reduced IPFP volume in end-stage OA patients [30], negatively associated with serum MMP-13, a metalloproteinase associated with inflammation and structural alterations [31]. While no differences were found between IPFP volume from OA and healthy individuals, nor association with pain [32–34]. IPFP depth, measured as the IPFP extension from anterior to posterior or thickness [35, 36], provided contradictory results [30, 36–38]. The IPFP area is obtained by drawing disarticulation contours around the boundaries, section by section [27]. IPFP maximal area was lower in OA patients [38], negatively associated with rOA [39] and femorotibial OA [40].

#### Signal intensity

IPFP displays hyperintense and hypointense signals under MRI analysis that are assessed through different semiquantitative scoring methods [27]. Hyperintense signals are the most frequently reported and often collectively referred to as IPFP signal intensity alterations [27]. Twelve cross-sectional studies described consistent correlations between IPFP signal intensity alterations and OA disease, including positive association with KL grading [31], symptomatic OA (sOA), rOA [36, 41], joint degeneration parameters, [33, 38], pain [42], biochemical biomarkers from serum of inflammation (interleukin-8 (IL-8), interleukin-17 (IL-17), resistin) and tissue structure alterations (MMP-13, ghrelin, and citrate levels) [31, 43–47].

According to Dragoo et al. [48], T2-hyperintense signals in IPFP have been related to inflammation and Hoffa synovitis because they correspond to blood vessels. By dynamic contrast-enhanced MRI (DCE-MRI) the assessment of tissue perfusion biomarkers in the hyperintense regions is possible [49]. Moreover, the degree of diffusion and/or perfusion is assessed by using intravoxel incoherent motion diffusion-weighted MR imaging (IVIM-DWI) parameters [50]. Findings from two studies showed increased perfusion and water diffusion in fat-suppressed T2 (T2FS) hyperintense regions in OA patients compared to healthy control subjects [51], and asymptomatic OA [36], respectively.

In addition, IPFP also shows hypointense signals observed as lower signal foci on T1- or T2-weighted MRI and are linked to fibrosis [48]. Three studies reported an increase of IPFP hypointense signal in the end-stage OA patients compared to moderate OA, and no-OA affected patients [30, 37], positively associated with rOA [52]. Fibrosis was evaluated in four studies by MRI (T2* relaxation time), ultrasound elastography (stiffness), 3D modeling (contracture), and fat fraction measurements. In this regard, high stiffness, contracture, and reduced fat content in OA IPFP were linked to anterior knee pain and OA severity suggesting an increase of IPFP fibrosis during OA progression [40, 53–55].

#### Prognostic value

A higher IPFP signal intensity and Hoffa synovitis has been correlated to the probability of developing end-stage OA and pain [56–60] in longitudinal studies, highlighting the possibility of quantifying hyperintense signals in IPFP to obtain prognostic biomarkers. Variations in the high IPFP signal intensity can be assessed by quantifying the mean, standard deviation, median, upper quartile, the volume of this signal, the ratio of the volume respect the whole IPFP, and the clustering regions with high signal intensity in IPFP. These measurements are known as Mean [H], sDev [H], Median [H], UQ [H], Volume [H], Percentage [H], and clustering factor [H] values, respectively [61]. Six studies revealed positive associations between these quantitative parameters and rOA, an incidence of TKA [61–64], and joint degeneration imaging for OA progression [65–67]. Particularly, sDev [H] and UQ [H], but not clustering factor [H], were additionally linked to biochemical markers of tissue turnover and inflammation [67].

Other approaches have been recently explored for IPFP analysis including MRI-texture scores, which consist of the quantification of voxel or pixel signal intensities allowing the study of tissue heterogeneity [68]. Results showed a higher discrimination and predictive value of incident rOA using 20 Voxel-based IPFP texture features (AUC ≥ 0.75) compared with clinical scores (AUC ≤ 0.69) [69]. Recently, two studies combined texture features, signal intensity, and geometric shape in a quantitative approach called radiomics**,** increasing the power of the decision support models [70]. Thus, the radiomic scores were positively associated with OA severity [71], and the combination of clinical and radiomic measurements provided a better OA diagnosis compared both parameters separately [72]. Finally, machine learning (ML) approaches were used to predict IPFP volume during OA progression [73].

#### Surgery outcome

Fibrosis affects the implant outcome after procedures including TKA or ACL reconstruction [74]. Shorter T2 values were found in individuals with severe scarring after TKA [74]. In this study, multiacquisition variable-resonance image combination (MAVRIC), technique that combines multiple individual image datasets acquired at incremented offsets of transmission and reception frequencies [75], was used to overcome implant interference. On the other hand, IPFP has been in debate because its routine resection during TKA may affect or not the joint functionality. The effect of IPFP resection was assessed by clinical, functional, and radiologic evaluation reporting inconclusive findings. Two studies found a worse isokinetic performance and patellar tendon shortening in complete IPFP resection compared to the preserved IPFP group [76, 77], whereas no differences were indicated in another study [78]. Longitudinal studies reported no significant alterations in patellar tendon and functional knee scores [79–81]. Results related to pain incidence were also contradictory [78, 80].

### Biochemical and molecular markers

The studies under this category included 230 OA patients and 146 control individuals **(**Table 2**)**. Five of fourteen articles reported differences due to age and /or BMI. Findings summarized in Table 2 showed that IPFP from OA patients consistently secreted and/or expressed higher levels of interleukin-6 (IL-6), adiponectin, and fatty acid-binding protein 4 (FABP4) [23, 82–84]. Other factors that were found elevated in OA patients compared to controls included adipokines and proteins related to lipid metabolism (chemerin, retinoic binding protein 4 (RBP4),WNT1 inducible signaling pathway protein 2 (WISP2), apolipoprotein (APO) A4, APOE), inflammatory (monocyte chemoattractant protein-1 (MCP-1), complement factor 8b (C8b), cluster of differentiation 68 (CD68)), matrix remodeling (cartilage oligomeric matrix protein (COMP), vitronectin (VTN), piezo1/2 mechanosensors, and yes1 associated transcriptional regulator (YAP1)), vascularization (vascular endothelial growth factor (VEGF), CD31, and CD34), and innervation (protein gene- product 9.5 or PGP9.5) [23, 85–89]. In contrast, a lower secretion of lymphotactin, collagen I (COL-I), and collagen III (COL-III) were found in the IFPF from OA patients compared to control IPFP obtained from arthroscopies [82, 83]. Findings related to leptin were contradictory [82, 86].

**Table 2:** Potential biochemical and molecular biomarkers in the IPFP from OA patients. AA: Arachidonic acid. AcCa: acylcarnitine. ACL: Anterior cruciate ligament. ACLR: Anterior cruciate ligament reconstruction. APOA4: Apolipoprotein A4. APOE: Apolipoprotein E. CD: Cartilage defect. Cer: Ceramide. COL-I: Collagen I. COL-III: Collagen III. COMP: Cartilage oligomeric matrix protein. FABP4: Fatty acid-binding protein 4. Hex-Cer: Hexosyl-ceramide. IH: Immunohistochemistry. IL-6: Interleukin-6. LC–MS: Liquid chromatography mass spectrometry. LysoPC: Lysophosphatidylcholine. MALDI-MSI: Matrix assisted laser desorption ionization – mass spectrometry imaging. MCP-1: Monocyte chemoattractant protein-1. PC: Phosphatidylcholine. PE Os: Ether-linked phosphatidylethanolamines. PGP9.5: Protein gene- product 9.5. PGE_2_: Prostaglandin E2. RBP4: Retinoic binding protein 4. TXB2: thromboxane B2. VEGF: Vascular endothelial growth factor. VTN: Vitronectin. WISP2: WNT1 inducible signaling pathway protein 2. XCL1: Lymphotactin. YAP1: Yes1 associated transcriptional regulator

Reference	Population	Approach	Findings
1) Belluzzi et al. [82]	End-stage OA (*n* = 25) Control (*n* = 28, ACL)	ELISA PCR Histology	IL-6, adiponectin, leptin, and FABP4 in OA group Adipocyte numbers, COL-I, COL-III
2) Favero et al. [23]	OA (*n* = 28) Control (*n* = 8, cadaver without OA signs)	Histology	VEGF, MCP-1, and IL-6
3) Wisniewska et al. [83]	OA (*n* = 9) Control (*n* = 12, arthroscopy)	Protein array	Adiponectin and XCL1 in the OA-IPFP
4) Zhang et al. [84]	OA (*n* = 38) Non-OA (*n* = 15, arthroscopic surgery)	ELISA	FABP4 in secretome of IPFP from OA patients
5) Conde et al. [85]	OA (*n* = 36) Control (*n* = 15, traumatic knee injury)	RT-PCR Western blot	WISP2
6) Grevenstein et al. [86]	End-stage OA (*n* = 14) Control (*n* = 11, ACLR)	Histology	COMP was detected in the fibrous zone in the IPFP Leptin in the OA group
7) Emmi et al. [87]	End-stage OA (*n* = 10) Control (*n* = 10, cadavers)	Histology	Piezo1/2 mechanosensors, CD68, PGP9.5 and YAP1 were expressed differently in the OA-IPFP compared to the control group
8) Tu et al. [88]	OA (*n* = 6) Control (*n* = 6, ACL)	LC–MS	APOA4, RBP4, C8B, and VTN in IPFP tissue Cer (d18:0/16:0) and HexCer (d18:1/34:0) AcCa (18:0), LysoPC (16:0), LysoPC (18:3), LysoPC (17:0), LysoPC (18:0), LysoPC (20:3)
9) Tang et al. [89]	OA (*n* = 9) Control (*n* = 4, organ donors)	IH	APOE expression in OA IPFP
10) Nieminen et al. [90]	End-stage OA (*n* = 10) RA (*n* = 10) Control (*n* = 5, arthroscopy)	Metabolomics	Testosterone sulfate, androsterone sulfate, cholest-4-en-26-oic acid, 7α-hydroxy-3-oxo, LysoPC (18:0), L-arginine, proline, glutamic acid, aspartic acid, L-pipecolic acid, histamine, 4-imidazoleacetic acid, guanidineacetic acid Low PC (16:0, 16:0)
11) Gierman et al. [91]	OA (*n* = 13) Control (*n* = 8, postmortem donors)	LC–MS	Conditioned media Lipoxin A4 TXB2 and AA
12) Timur et al. [92]	End-stage OA (*n* = 17) Control (CD, *n* = 12)	ELISA	PGE_2_ in secretome from high PGE_2_ OA group No differences between the low PGE_2_ OA group and the controls
13) Haartmans et al. [24]	End-stage OA (*n* = 7) Control (CD, (*n* = 7)	MALDI-MSI	IPFP fibrosis was found in OA patients PE O-s, containing AA in the connective tissue of the OA IPFP
14) Jiang et al. [93]	OA (*n* = 3) Control group (*n* = 3, ACLR)	RNA-seq	hsa_circ_0005265 in both synovium and IPFP

OA IPFP secreted and/or expressed higher levels of lysophosphatidylcholine (lysoPC) species [88, 90]. Other lipid mediators and metabolites increased in OA IPFP included thromboxane B2 (TXB2), prostaglandin E_2_ (PGE_2_), arachidonic acid (AA) [98], amino acids (L-arginine, proline, glutamic acid, aspartic acid, L-pipecolic acid, histamine, 4-imidazole acetic acid, and guanidine acetic acid), steroids (testosterone sulfate, androsterone sulfate), and bile metabolites (cholest-4-en-26-oic acid, 7α-hydroxy-3-oxo) [90–92]. Similarly, a higher presence of ether-linked phosphatidylethanolamines (PE O-s) containing AA in the connective tissue of OA IPFP compared to those from patients suffering cartilage defects revealed by Matrix-assisted laser desorption/ionization mass spectrometry imaging (MALDI-MSI) [24]. On the other hand, lower levels of lipoxin A4, phosphatidylcholine (PC), and ceramide metabolites (Cer (d18:0/16:0) and HexCer (d18:1/34:0) in the OA group were also reported [88, 91]. Finally, a lower expression level of circular RNA (circRNA) hsa_circ_0005265 for both IPFP and synovium from OA patients with respect to ACL control individuals has been described [93].

## Discussion

Early diagnosis and effective treatment for OA are still unmet needs. It is urgent to improve the OA clinical diagnosis not only in terms of early detection but also in predicting the risk for early onset and rapid progression. This could guide the application of joint preserving treatments according to OA-specific endo/phenotypes. Recent evidence indicates that IPFP is an active player in OA progression; however, the molecular mechanisms involved in the OA context remain unclear. Compared to other compartments in the knee, including cartilage and synovium, IPFP has been less studied even though its availability since it is commonly removed as waste material during orthopedic surgeries [24]. For this reason, this review aimed to investigate the potential of the IPFP as a novel source of biomarkers and therapeutic targets for OA.

**Fig. 3:**
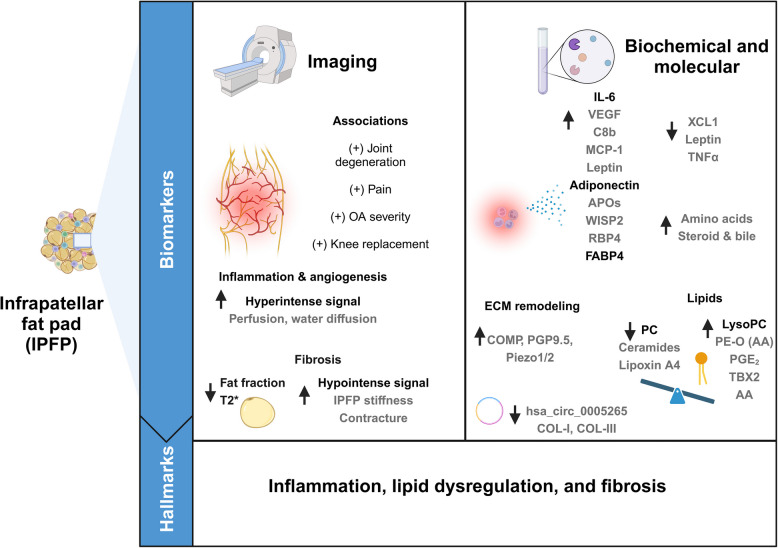
Summary of potential imaging, biochemical, and molecular biomarkers reported in the IPFP for OA disease. Imaging-based studies indicated the positive association of hyperintense signal alterations in IPFP with inflammation, angiogenesis, joint degeneration parameters, OA progression, and knee replacement. Moreover, higher hypointense signals in IPFP, lower fat fraction and T2 relaxation times (T2*) suggested fibrosis. On the other hand, biochemical and molecular studies showed increased levels of IL-6, adiponectin, FABP4, and lysoPC in OA IPFP. Alterations in the expression/secretion of cytokines, chemokines, adipokines, apolipoproteins (APOs), tissue structural components, lipids, amino acids, AA, steroids, TBX2, PGE_2_, and bile molecules were also reported. Parameters or molecules that were detected in one study or with controversial results are displayed in grey. These findings suggest the implication of inflammation, lipid dysregulation, and fibrosis of the IPFP in OA pathology. Created with Biorender

Most research on IPFP was oriented to imaging biomarkers. IPFP was mostly assessed through MRI, offering the advantage of including a higher number of patients and enabling comparisons with healthy individuals, and those adjusted by age, gender, and BMI. While MRI morphological parameters like IPFP volume provided controversial results, signal alterations were more consistent. In fact, IPFP signal imaging semiquantitative alterations have revealed inflammation and angiogenesis in the IPFP from OA patients that were positively correlated with OA progression, joint degeneration imaging parameters, and pain (Fig. 3). Moreover, quantitative imaging parameters such as sDev [H] and UQ [H], were consistently linked to biochemical markers of tissue turnover and inflammation. Furthermore, the combination of radiomic and clinical data showed better prognostic performances compared to both separately. A similar approach, combining texture analysis, radiomics, and ML approaches exhibited good prognostic performances for subchondral bone assessment [94].

Regarding biochemical and molecular-based studies, high levels of IL-6, lysoPC, FABP4, and adiponectin**,** were consistently observed in OA patients with respect to control individuals. These molecules are typically associated with inflammation and lipid metabolism (Fig. 3). Similarly, a proteomic study of the IPFP secretome also showed the upregulation of complement factors 3 and 5 (C3, C5), proteins related to lipid metabolism (perilipin 4 (PLIN4), and apolipoprotein (APOB-100)) [95]. Particularly, IL-6 has been associated not only with cartilage loss but also with pain playing a key role in OA worsening [96]. Likewise, a higher expression of complement factor 3a (C3a) and C5b has been reported in synovial fluid from early OA patients [97].

During the inflammation the conversion of PC into lysoPC by phospholipase A2 (PLA2) also occurs [98]. Higher lysoPC and AA levels as well as a lower level of PC are consistent with previous studies on plasma and serum from OA patients [98–100]. In a similar line, lipid-associated protein FABP4 has been negatively associated with cartilage thickness in end-stage OA patients [101]. Recently, FABP3 and phospholipase A2 group IIA (PLA2G2A) were upregulated in the IPFP proteome of patients suffering from cartilage defects with worse knee functionality and pain [102], revealing the link between lipid dysregulation and pain in individuals with high risk of developing OA. Together, these findings indicate that lysoPC and FABP4 could be potential biomarkers in the IPFP for OA disease whereas lipid-related proteins represent intriguing targets for future research.

The role of adiponectin in OA is currently debated due to some studies indicating that adiponectin exhibits a catabolic effect on cartilage, modulates its degradation, or is even associated with OA severity [103, 104]. Serum adiponectin levels were associated with OA but were negatively correlated with IL-6 and C-reactive protein in knee OA. In contrast, it decreased in obese patients with poor physical performance whereas IL-6 remained higher [105]. Similarly, higher leptin and lower adiponectin gene expressions were found in the obese group compared to the non-obese group [106, 107]. Considering that, adiponectin regulates glucose and lipid metabolism, reduces glucose, and increases fatty-acid oxidation [108]. Higher adiponectin levels could serve as a protective mechanism to manage lipid metabolism and inflammation during OA progression; however, it is reduced due to metabolic imbalance in obesity and diabetic scenarios, which have been proposed that aggravate OA [109]. Alterations in lipid metabolism and metabolic syndrome have been implicated in OA [98, 109, 110]. A recent review described the interplay between obesity, adipose tissue dysfunction, and metabolic syndrome in OA disease and pain [111]. Lipodystrophy mouse models showed that systemic adipose tissue dysfunction may induce loss of articular cartilage homeostasis mediating joint degeneration in cooperation with alteration of intra-articular adipose tissue [112].

Furthermore, an increase in several apolipoprotein levels in the IPFP may be related to a compensatory mechanism to overcome lipid dysregulation during OA. Synovial APOA1 and serum APOB-100 levels have been negatively associated with cartilage damage, and radiographic and symptomatic OA [113]. Little is known regarding APOE levels in OA patients. Transcriptomic analyses revealed an increase of APOE signaling in IPFP related with deleterious effects in a murine collagenase-induced OA model [89] whereas APOE knockdown caused OA in mice [114]. Thus, further studies regarding the adipokine and apolipoprotein levels in the IPFP in the OA context are needed.

Alongside inflammation and lipid dysfunction, a growing body of evidence through different imaging, biochemical, and molecular parameters were indicators of fibrotic processes and pain in the OA IPFP (Fig. 3). Hypointense signals, T2 relaxation values in the IPFP, and its fat fraction allowed fibrosis assessment, indicating their suitability as fibrotic biomarker. Transcriptomic analyses revealed differences in cell adhesion and integrin signaling pathways between OA and healthy IPFP [115]. These changes along with the histopathological changes in the IPFP during OA [22], could be related to IPFP fibroblasts phenotype towards a fibrotic version. Importantly, this transcriptomic analysis also showed that joint lubricating mechanisms by IPFP fibroblasts can be reduced in obese OA individuals highlighting the relevance of IPFP function in biomechanical terms for knee joints [115].

Currently, it is still debated whether the IPFP displays protective or degenerative roles, or it should be resected or preserved during knee surgeries. According to a recent review, IPFP displays both roles in OA progression and there is no consensus on the decision to resect or preserve the IPFP [116]. Considering the studies included in this review, IPFP might suffer different changes that contribute to inflammation and fibrosis, linked to pain and OA progression. IPFP may possibly exhibit a protective role not only due to the presence of MSC but also through the potential biomechanic role, maintenance of metabolic and antioxidant balance. Nevertheless, studies that evaluated the surgery outcome after TKA offered inconclusive results. This is probably due to technical differences (radiography and clinical evaluation) and lower number of participants compared to cross-sectional and longitudinal studies performed before TKA, introducing an increased heterogeneity by BMI, age, and gender.

### Limitations

In this review, we found several limitations in the current literature of IPFP as a source of potential OA biomarkers. Most imaging-based articles used only KL grading to classify OA severity. In the future, the classification of OA might be more robust if it includes MRI scores such as MRI osteoarthritis knee score (MOAKS), Boston Leeds osteoarthritis knee score (BLOKS), and WORMS, which better reflect the knee structure abnormalities. Moreover, the imaging-based studies were heterogeneous in terms of OA definition and classification encouraging the OA community to make more efforts into it. Despite promising findings regarding quantitative imaging biomarkers, further studies are needed to investigate their association with biochemical parameters from local tissues in longitudinal studies to build more precise diagnostic and prognostic models.

Unlike cross-sectional and longitudinal imaging studies performed before TKA, those studies investigating the surgery outcome included a low number of patients with limited quality, considering the potential application of more advanced imaging techniques to describe the post-TKA effects. These aspects made impractical drawing conclusions related to the resection-preservation clinical debate. Regarding biochemical and molecular biomarkers, we found four main drawbacks in this category: 1) a limited number of studies, 2) limited sample size, 3) control and OA groups showed differences due to age and BMI, and 4) a lack of healthy controls. Some reports regarding chemokines and adipokines were not conclusive, possibly due to differences in detection technique (RNA vs. protein), patient variability, or their biological roles. These disadvantages, especially the presence of only one study, did not allow us to draw strong conclusions on the potential biochemical and/or molecular biomarkers for fibrosis.

### Future directions and considerations

Imaging-based studies of IPFP have offered clues to OA progression. Nowadays, the implementation of low-field MRI increases the availability of MRI analysis at lower costs [117]. Even though the inferior resolution of low-field MRI, there are several approaches to mitigate this disadvantage, including the support with artificial intelligence (AI) or deep learning tools [117, 118]. Other techniques including ultrasound and ^1^H MRS may support the IPFP assessment. Interestingly, MALDI-MSI approach also provided the visualization of potential lipidic biomarkers involved in inflammation and their spatial distribution in the IPFP enabling them to address the intra-tissue heterogeneity [119]. Then, this technique could be also combined with MRI to support the study of OA in pre/early and mild/moderate stages. Therefore, the IPFP study could not only provide insights into understanding its role in OA but also provide novel imaging and biochemical biomarkers. The combination of MRI assessments and multiomic profiles of local tissues could also contribute to the discovery of novel biomarkers and unveiling signaling pathways. More comprehensive diagnostic methods could use ML methodologies for the integration of biomarker levels, clinical, and demographic variables.

Further biochemical and molecular studies exploring adipokines, secretory profiles, including exosomes, and regulatory molecules such as circRNAs and miRNAs, could lead to the identification of novel potential biomarkers. Moreover, differences in adipose/connective ratio within the IPFP might also explain inconsistencies observed in molecular studies**.** Therefore, by using high throughput technologies including single-cell, single-nuclei RNA sequencing as well as spatial proteomics it is possible to elucidate which cell populations are responsible for the differential molecular profiles in IPFP.

In addition, IPFP can be proposed as a source to study patient heterogeneity and to investigate different OA endotypes. OA endo/phenotypes change over time due to gaining weight, trauma, medication use, and losing or increasing activity. In vitro explant-based models could represent a tool to closely recapitulate the microenvironment at different stages of joint disease. Menisci, ligaments, and other tissues can also be incorporated into microchips enabling the study of not only the inflammatory and/or biomechanical stimuli but also the interaction between different joint tissues. Such approaches may be valuable in revealing not only potential endotype-associated biomarkers but also the underlying molecular mechanisms associated with OA. These novel technologies could allow us to gain a deep insight into the modulation potential targets for further personalized medicine approaches.

## Conclusions

Imaging, biochemical, and molecular studies reveal that IPFP undergoes critical events associated with OA, including inflammation, angiogenesis, and fibrosis, that were linked to OA progression and pain. In this regard, IPFP could be considered a source of OA biomarkers that also provide insights into its pathophysiology. Remarkably, higher levels of IL-6, FABP4, adiponectin, and lysoPC suggest that IPFP could contribute to OA progression due not only to an imbalance between pro- and anti-inflammatory mediators but also through dysregulation of lipid metabolism. Potential protective mechanisms against lipid alterations could be disrupted in obese and diabetic patients. However, further research is needed to address these possible associations. Imaging parameters and emerging molecular evidence indicated the link between IPFP fibrosis during OA demanding further investigations into biomechanical effects. Therefore, more research into IPFP, particularly high throughput studies involving larger patient cohorts, and the investigation of IPFP profile (secretome, proteome, metabolome, extracellular vesicles, RNAs). Notably, the combination of several imaging and biochemical biomarkers along with ML methods could offer an efficient diagnosis. These efforts could lead to the discovery of novel biomarkers, enabling an earlier diagnosis; and supporting a better OA patient stratification by molecular endotypes to tailor treatment for future precision medicine.

## Supplementary Information

Supplementary Material 1.

## Data Availability

No datasets were generated or analysed during the current study.

## Abbreviations

AA: Arachidonic acid
ACL: Anterior cruciate ligament
ACLR: Anterior cruciate ligament reconstruction
ACR: American College of Rheumatology
APOs: Apolipoproteins
APOA1: Apolipoprotein A1
APOA4: Apolipoprotein A4
APOB-100: Apolipoprotein B-100
APOE: Apolipoprotein E
AUC: Area under curve
BLOKS: Boston Leeds osteoarthritis knee score
BMI: Body mass index
CD: Cartilage defect
CD31: Cluster of differentiation 31
CD34: Cluster of differentiation 34
CD68: Cluster of differentiation 68
Circular RNAs: CircRNAs
Clustering factor [H]: Clustering regions with high (hyperintense) signal intensity in the IPFP
COL-I: Collagen type I
COL-III: Collagen type III
COMP: Cartilage oligomeric matrix protein
C3: Complement factor 3
C5: Complement factor 5
C3a: Complement factor 3a
C5b: Complement factor 5b
C8b: Complement factor 8b
CSA: Cross-sectional area
DCE-MRI: Dynamic contrast-enhanced MRI
ECM: Extracellular matrix
ECs: Endothelial cells
FABP4: Fatty acid-binding protein 4
FSE: Fast spin echo
KOOS: Knee injury and osteoarthritis outcome score
IH: Immunohistochemistry
IL-6: Interleukin-6
IL-8: Interleukin-8
IL-17: Interleukin-17
IPFP: Infrapatellar fat pad
IPFP [H]: High (hyperintensity) IPFP signal intensity alteration
IVIM-DWI: Intravoxel incoherent motion diffusion-weighted MR imaging
JNS: Joint space narrowing score
KL: Kellgren lawrence
LC-MS: Liquid chromatography mass spectrometry
LysoPC: Lysophosphatidylcholine
MALDI-MSI: Matrix assisted laser desorption ionization – mass spectrometry imaging
MAVRIC: Multiacquisition variable-resonance image combination
MCP-1: Monocyte chemoattractant protein-1
Mean [H]: Mean value of high (hyperintense) IPFP signal intensity
Median [H]: Median value of high (hyperintense) IPFP signal intensity
MEDIC: Multi-echo data image combination
ML: Machine learning
MMP13: Metalloproteinase 13
MOAKS: MRI osteoarthritis knee score
MRI: Magnetic resonance imaging
MSCs: Mesenchymal stromal cells
NCs: Neuronal cells
OARSI: Osteoarthritis research society international atlas scale
OA: Osteoarthritis
Percentage [H]: Ratio of the volume [H] respect the whole high (hyperintense) IPFP signal intensity
PFJ: Patellofemoral joint
PLIN4: Perilipin 4
PLA2: Phospholipase A2
PLA2G2A: Phospholipase A2 group IIA
PC: Phosphatidylcholine
PE O-s: Phosphatidylethanolamines
PGP9.5: Protein gene- product 9.5
PGE_2_: Prostaglandin E_2_
rOA: Radiographic OA
RBP4: Retinoic binding protein 4
sDev [H]: Standard deviation value of high (hyperintense) IPFP signal intensity
SGE: Spoiled gradient echo
SPAIR: Spectral attenuated inversion recovery
SPGR: Spoiled gradient recall
SWE: Ultrasound shear elastography
sOA: Symptomatic OA
TSE: Turbo spin echo
TKA: Total knee arthroplasty
TBX2: Thromboxane B2
T1-w: T1-weighted MRI
T2FS: Fat-suppressed T2 hyperintense regions
T2-w: T2-weighted MRI
T2*: T2 relaxation times
UQ [H]: Upper quartile value of high (hyperintense) IPFP signal intensity
VEGF: Vascular endothelial growth factor
VTN: Vitronectin
Volume [H]: Volume value of high (hyperintense) IPFP signal intensity
WISP2: WNT1 inducible signaling pathway protein 2
WOMAC: Western Ontario and McMaster universities osteoarthritis index
WORMS: Whole-organ MRI score
XCL1: Lymphotactin
YAP1: Yes1 associated transcriptional regulator
^1^H-MRS: Hydrogen proton magnetic resonance spectroscopy
