# Supplementary material for: Infrapatellar fat pad as a source of biomarkers and therapeutic target for knee osteoarthritis
Source: Arthritis Res Ther. 2025 Apr 5;27:81. doi: 10.1186/s13075-025-03517-8 (PMC11972505; doi:10.1186/s13075-025-03517-8)
Supplement: Supplementary file 1 — Supplementary Material 1. [file 13075_2025_3517_MOESM1_ESM.docx]

**Supplemental table 1. PubMed advanced search conducted on June 4^th^, 2024.** Thirteen queries were used giving a total of 1387 records. Then, records were stored in a NCBI providing a collection of 474 records.

| **Search** | **Query** | **Items found** |
| --- | --- | --- |
| 1 | ((infrapatellar fat pad) OR (hoffa fat pad)) AND (osteoarthritis) | 381 |
| 2 | ((infrapatellar fat pad) OR (hoffa fat pad)) AND (biomarker) | 73 |
| 3 | ((infrapatellar fat pad) OR (hoffa fat pad)) AND (imaging biomarker) | 34 |
| 4 | ((infrapatellar fat pad) OR (hoffa fat pad)) AND (imaging) | 410 |
| 5 | ((infrapatellar fat pad) OR (hoffa fat pad)) AND (magnetic resonance imaging) | 305 |
| 6 | ((infrapatellar fat pad) OR (hoffa fat pad)) AND (molecular biomarker) | 10 |
| 7 | ((infrapatellar fat pad) OR (hoffa fat pad)) AND (mass spectrometry) | 9 |
| 8 | ((infrapatellar fat pad) OR (hoffa fat pad)) AND (proteomics) | 11 |
| 9 | ((infrapatellar fat pad) OR (hoffa fat pad)) AND (metabolomics) | 6 |
| 10 | ((infrapatellar fat pad) OR (hoffa fat pad)) AND (lipidomics) | 5 |
| 11 | ((infrapatellar fat pad) OR (hoffa fat pad)) AND (gene expression) | 98 |
| 12 | ((infrapatellar fat pad) OR (hoffa fat pad)) AND (RNA) | 45 |
| 13 | ((infrapatellar fat pad) OR (hoffa fat pad)) AND (imaging biomarker) | 34 |
